# SLXL1, a Novel Acrosomal Protein, Interacts with DKKL1 and Is Involved in Fertilization in Mice

**DOI:** 10.1371/journal.pone.0020866

**Published:** 2011-06-15

**Authors:** Xin-jie Zhuang, Xiao-jun Hou, Shang-Ying Liao, Xiu-Xia Wang, Howard J. Cooke, Ming Zhang, Chunsheng Han

**Affiliations:** 1 State Key Laboratory for Conservation and Utilization of Subtropical Agro-bioresources, Animal Reproduction Institute, Guangxi University, Nanning, Guangxi, People's Republic of China; 2 State Key Laboratory of Reproductive Biology, Institute of Zoology, Chinese Academy of Sciences, Beijing, People's Republic of China; 3 Institute of Genetic and Molecular Medicine MRC Human Genetics Unit, Western General Hospital, Edinburgh, Scotland, United Kingdom; Clermont Université, France

## Abstract

**Background:**

Spermatogenesis is a complex cellular developmental process which involves diverse families of genes. The Xlr (X-linked, lymphocyte regulated) family includes multiple members, only a few of which have reported functions in meiosis, post-meiotic maturation, and fertilization of germ cells. Slx-like1 (Slxl1) is a member of the Xlr family, whose expression and function in spermatogenesis need to be elucidated.

**Methodology/Principal Findings:**

The mRNA and protein expression and localization of Slxl1 were investigated by RT-PCR, Western blotting and immunohistochemistry in different tissues and at different stages of spermatogenesis. The interacting partner of SLXL1 was examined by co-immunoprecipitation and co-localization. Assessment of the role of SLXL1 in capacitation, acrosome reaction, zona pellucida binding/penetration, and fertilization was carried out *in vitro* using blocking antisera. The results showed that Slxl1 mRNA and protein were specifically expressed in the testis. SLXL1 was exclusively located in the acrosome of post-meiotic germ cells and interacts with DKKL1 (Dickkopf-like1), which is an acrosome-associated protein and plays an important role in fertilization. The rates of zona pellucida binding/penetration and fertilization were significantly reduced by the anti-SLXL1 polyclonal antiserum.

**Conclusions/Significance:**

SLXL1 is the first identified member of the XLR family that is associated with acrosome and is involved in zona pellucid binding/penetration and subsequent fertilization. These results, together with previous studies, suggest that Xlr family members participate in diverse processes from meiosis to fertilization during spermatogenesis.

## Introduction

Spermatogenesis is a complex process that can be divided into three stages: mitosis in spermatogonia, meiosis in spermatocytes, and spermiogenesis in round spermatids and later cell types. The end products of spermatogenesis are the spermatozoa that contain specialized structures such as flagellum and acrosome. The acrosome plays an essential role during fertilization and is formed from a secretory vesicle by a Golgi-derived apparatus in the initial steps of spermiogenesis [1]. Acrosome biogenesis is a multi-step process including vesicular trafficking and organelle migration [1], [2], [3]. The acrosome contains a variety of proteins including protease zymogens, zona pellucida-binding proteins and DKKL1 protein [4], [5], [6], [7]. Lack of acrosome reduces the rate of fertilization *in vitro* [8], [9]. Acrosome reaction is an exocytotic event in fertilization that releases enzymes to facilitate the spermatozoon's binding to and penetration of the zona pellucid [10]. Clinical studies have identified patients whose infertility is associated with an abnormal acrosome reaction [11].

The expression of Xlr (X-linked lymphocyte-regulated complex) genes was initially identified in terminally differentiated B lymphoid cells [12], [13]. The proteins of this family, sharing the COR1 domain, include XLR, SYCP3 (Synaptonemal complex protein 3), SLY (Sycp3-like, Y-linked), and SLX/XMR (Sycp3-like, X-linked). XLR is the prototype member, and is a nuclear protein expressed in the spermatocyte and oocyte nucleus during prophase of meiosis [14]. SYCP3 is expressed in meiotic cells and is a basic component of the lateral elements of the synaptonemal complex [15]. Sycp3 is required for male fertility and the Sycp3^−/−^ male germ cells die around the zygotene stage of meiosis [16], [17]. Sycp3-deficient females are fertile but the embryos are not viable due to massive aneuploidy [18], [19]. Sly, a Y chromosome-linked gene of the family is only expressed in spermatids during spermatogenesis [20]. Interestingly, SLY interacts with DKKL1 which is involved in fertilization [20] probably by facilitating spermatozoon's penetration of the zona pellucida [8], [9]. In Sly-deficient mice, spermatozoon differentiation is severely impaired, and the spermatozoon heads are abnormal [21]. Slx/Xmr is an X-linked gene and its protein shares 94% and 43% homologies with Xlr and Sly, respectively [22]. Slx/Xmr was initially reported to be a nuclear protein expressed during meiosis [23], [24]. Another study indicated that Slx encodes a spermatid cytoplasmic protein [25]. Moreover, the spermatozoa of Slx-deficient mice displayed abnormal heads and the males were sterile [26]. Slx-like1 (Slxl1) is a multi-copy gene (∼16 copies) located on the murine×chromosome [27]. SLXL1 and SLX expression were up-regulated in Sly-deficient mice [26]. However, the expression and function of SLXL1 in spermatogenesis have not been well-addressed. In the present study, we conducted an in-depth investigation of the expression of Slxl1 at both the mRNA and the protein levels. We report that Slxl1 protein is specifically present in the acrosomes of all post-meiotic germ cells. In addition, SLXL1 interacts with DKKL1 and plays an important role in zona pellucida binding/penetration and fertilization. Our results together with previous ones by others indicate that the XLR family proteins may be involved in diverse processes from meiosis to fertilization during spermatogenesis.

## Results

### Slxl1 was a novel testis-specific member of the mammalian Xlr superfamily

**Figure 1:**
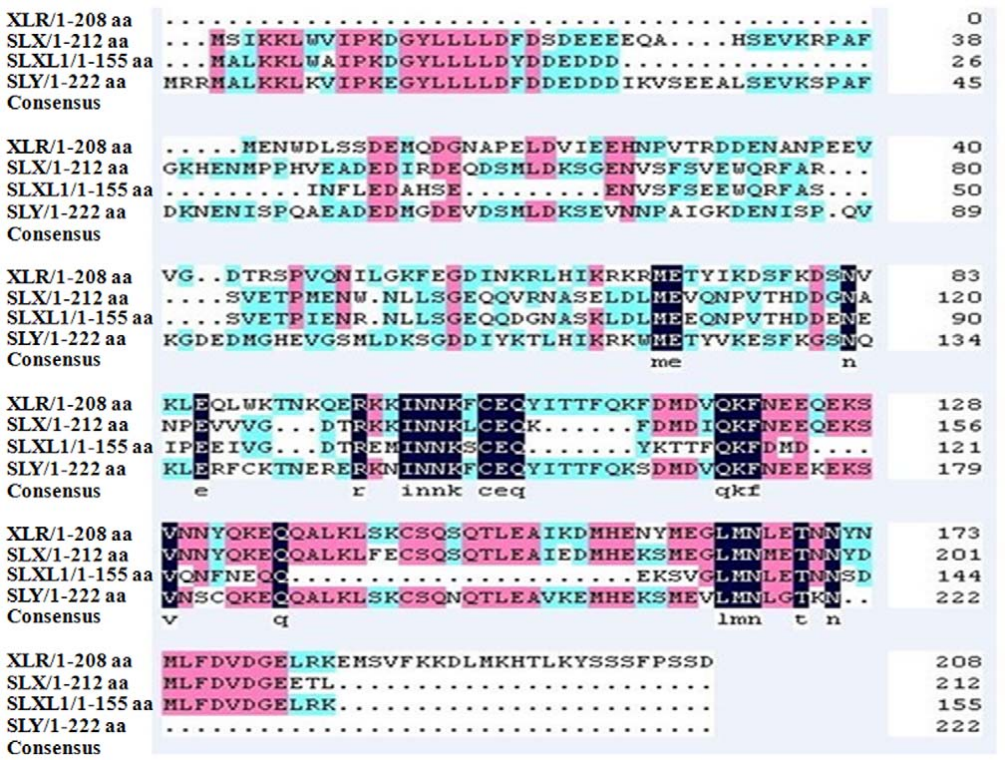
Amino acid sequence alignment of several representative members of the XLR family.

Slxl1 was initially identified as one of the large number of testis-specific genes by mining microarray expression data (unpublished data). The protein (155 aa) was annotated by the NCBI Conserved Domain Database to belong to the XLR superfamily, whose members share the COR1 domain. SLXL1 does not contain additional domain(s)/motif(s) such as signal peptide, transmembrane region(s) indicating it is most likely a cytoplasmic protein. Its closest paralogs in the mouse genome are SLX and SLY with identity of 69% and 42% to SLXL1, respectively (Fig. 1).

**Figure 2:**
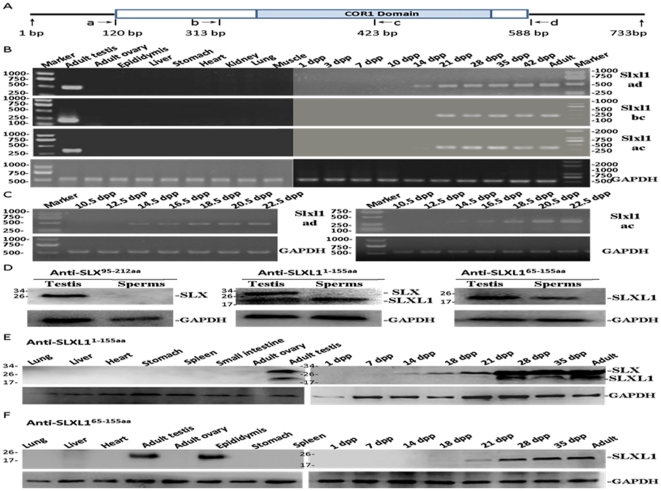
mRNA and protein expression of Slxl1 in mouse tissues. (A). Positions of primers used in RT-PCRs. (B) Slxl1 mRNA was exclusively expressed in mouse testes indicated by RT-PCR analysis of multiple tissues; Slxl1 transcript was first detected at 14 days post partum (dpp) in postnatal mouse testes. (C) From 10.5 dpp to 22.5 dpp, Slxl1 transcript level increased gradually as detected by RT-PCR using the ad and ac primer sets. (D) One protein of about 25 kDa in adult testis was detected in Western blotting, however, the protein was not detected in the spermatozoa using anti-SLX^95–212aa^ antibody. Using anti-SLXL1^1–155aa^, two proteins of about 25 kDa and 18 kDa were detected in testes, while only the 18 kDa one was detected in spermatozoa. Only the 18 kDa protein was detected in either testis or spermatozoa using anti-SLXL1^65–155aa^. (E–F) The expression of SLX and SLXL1 in mouse tissues and testes of different days post partum were detected using anti-SLXL1^1–155aa^ (E) and anti-SLXL1^65–155aa^ (F). mRNA and protein expression of GAPDH were used as internal controls for RT-PCR and Western Blot, respectively.

The expression of Slxl1 mRNA was detected by RT-PCR using three pairs of primers (Fig. 2A) in various tissues of mice. Slxl1 mRNAs was only detected in the testis (Fig. 2B). Moreover, Slxl1 mRNA expression in testis was weakly detected at 14 dpp (day post partum) when spermatogenesis progressed to the pachytene spermatocyte stage, and was greatly increased at 21 dpp and thereafter when spermatids are produced in the first wave of spermatogenesis [28]. To examine the expression in a more precise manner, additional time points, one every two days from 10.5 dpp to 22.5 dpp, were included for the RT-PCR detection. As shown by Fig. 2C, the Slxl1 mRNA was first detected at 14 dpp and increased it level gradually until 22.5 dpp.

In order to study the protein expression of SLXL1 by immunostaining, two rabbit polyclonal antisera (anti-SLXL1^1–155aa^ and anti-SLXL1^65–155aa^) were developed using the GST-fusion proteins of full-length SLXL1 and the C-terminal fragment, respectively. A similar antiserum against the C-terminal part of SLX (anti-SLX^95–212aa^) was also produced. The predicted molecular mass of SLX and SLXL1 are 25 kDa and 18 kDa respectively. As shown in Fig. 2D, the anti-SLX^95–212aa^ in Western blotting detected a protein of about 25 kDa in adult testis but not in mature spermatozoa, indicating that this antiserum specifically recognizes SLX. While anti-SLXL1^1–155aa^ in Western blotting detected two proteins of about 25 kDa and 18 kDa in the testis, it only detected the 18 kDa one in the spermatozoa, indicating that this antiserum recognizes both SLX and SLXL1 when both are present. In contrast, the anti-SLXL1^65–155aa^ only detected SLXL1 in both testis and spermatozoa. The SLXL1 was expressed only in the testis of the several mouse tissues examined using both anti-SLXL1^1–155aa^ and anti-SLXL1^65–155aa^ (Fig. 2E and 2F). Consistent with the results of a previous study [25] and of the Slxl1 mRNA expression in the present study, SLXL1 was dramatically up-regulated post-meiotically starting from a time point between 21 and 28 dpp.

### SLXL1 was an acrosome-associated protein

**Figure 3:**
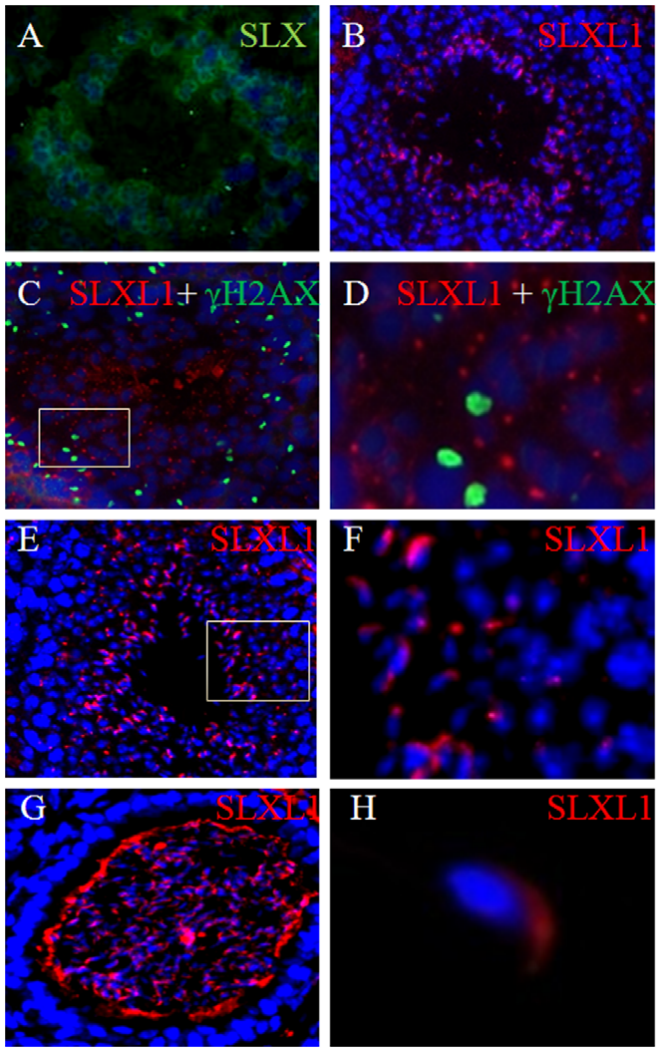
Localization of SLXL1 in the testis and mature spermatozoa. (A) Co-staining of adult testis sections for the DAPI(blue)and SLX (green) using anti-SLX^95–212aa^; (B) Immunostaining of adult testis sections using anti-SLXL1^1–155aa^; (C–D) A Stage VIII tubule immunostained by antisera for SLXL1 (red) and γH2AX (green); (E–F) A Stage VI-VII tubule showing the SLXL1 (red) localization in the round spermatid and elongating spermatozoa. (G) Immunostaining of SLXL1 in the spermatozoa of epididymis (red) showing its localization in the acrosome. (H) SLXL1 (red) was present in the acrosome of mature spermatozoa. D and F are the magnified views of the rectangular areas in C and E, respectively.

**Figure 4:**
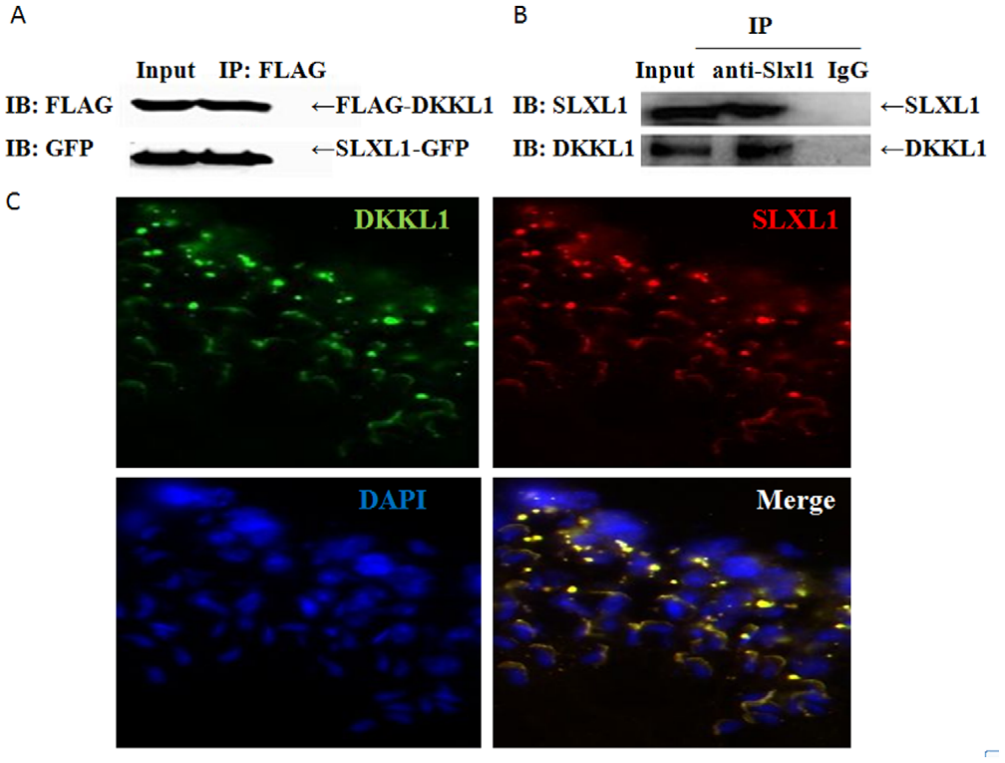
Co-immunoprecipitation and co-localization of SLXL1 and DKKL1. (A). Coimmunoprecipitation of DKKL1-FLAG with SLXL1-GFP expressed in 293T cells. (B) Coimmunoprecipitation of DKKL1 with SLXL1 from testis lysates. Protein lysates were analyzed by standard Western blotting with anti-FLAG, anti-GFP, anti-SLXL1 and anti-DKKL1 antisera, respectively. (C) Co-localization of SLXL1 (red) and DKKL1 (green) in seminiferous tubules. The nuclei were stained with DAPI (blue).

To examine the cellular and sub-cellular localization of SLXL1 protein in testis, immunostaining was performed on adult testis sections using anti-SLXL1^1–155aa^ (anti-SLXL1^65–155aa^ failed to work immunohistochemically). Small bright SLXL1-positive particles adjacent to the nuclei of post-meiotic germ cells were detected, and this type of signal was in strong contrast to that of SLX which was diffusely cytoplasmic in post-meiotic germ cells (Fig. 3A and 3B). The change in shape of the SLXL1 signal from round to oval/crescent particles as spermatids progress from round to elongated ones suggested that SLXL1 could be localized to acrosomes of different developmental stages (Fig. 3C–3F). Indeed, SLXL1 co-localized with an acrosomal protein DKKL1 at these stages (Fig. 4C). Moreover, the SLXL1 particles did not co-localize with γH2AX (phosphorylated H2A histone family, member X) (Fig. 3C), a marker for DNA double-strand break repair and the formation of sex body during meiosis of spermatocytes [29]. The acrosome localization of SLXL1 was most evident in released spermatozoa in the epididymis as the acrosomes can be easily recognized by its crescent shape on the apex of spermatozoa (Fig. 3G and 3H).

### SLXL1 interacted with acrosomal protein DKKL1

Since both SLXL1 and DKKL1 are localized in the acrosome and SLY, a close paralog of SLXL1 interacts with DKKL1, we wondered whether SLXL1 and DKKL1 interact with each other. This turned out to be the case as indicated by co-immunprecipitation (Co-IP) results. First, SLXL1-GFP and DKKL1-FLAG proteins expressed in K293T cells co-immunoprecipitated with each other (Fig. 4A). Second, the two native proteins in testis lysate also interacted with each other when the anti-SLXL1 and anti-DKKL1 antisera were used for IP and IB, respectively (Fig. 4B). Consistent with this, SLXL1 and DKKL1 co-localized in round and elongating spermatids (Fig. 4C).

### In vitro fertilization was reduced by SLXL1 antiserum

**Figure 5:**
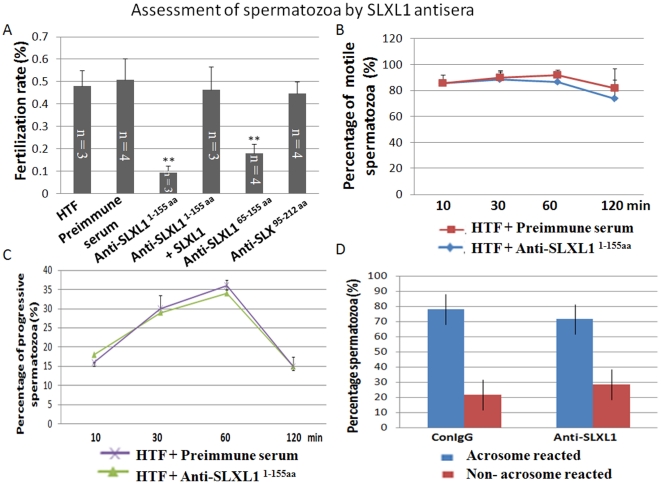
Assessment of the effects of SLXL1 antisera on *in vitro* fertilization, motility and acrosome reaction of spermatozoa. (A) The inhibitory effect of SLXL1 antisera on *in vitro* fertilization (IVF) rate. Successful fertilization was indicated by zygote cleavage. Spermatozoa were treated with PBS, preimmune serum, anti-SLXL1^1–155aa^, anti-SLXL1^1–155aa^+SLXL1, anti-SLXL1^65–155aa^ and anti-SLX^95–212aa^ before *in vitro* fertilization assays were performed. (B). Assessment of effect of anti-SLXL1^1–155aa^ on the motility of spermatozoa. (C). Assessment of effect of anti-SLXL1^1–155aa^ on the progressive movement of spermatozoa. (D). Assessment of effect of anti-SLXL1^1–155aa^ on the acrosome reaction of spermatozoa. **Denotes group that is significantly different (p<0.01) from the control groups.

We used *in vitro* fertilization assays to investigate a possible role for SLXL1 in fertilization. Successful *in vitro* fertilization was identified by the appearance of embryos at the 2-cell and 4-cell stages. While the fertilization rates of the untreated group, the pre-immune serum treated group, and the anti-SLX^95–212aa^ treated group are all above 45%, that of the anti-SLXL1^1–155aa^ and anti-SLXL1^65–155aa^ treated groups dropped significantly to 8% and 17%, respectively (Fig. 5A). To exclude the possibility that the antiserum itself may contain any toxic factor to fertilization, we added recombinant GST-SLXL1 protein to the anti-SLXL1^1–155aa^ treated group and the fertilization rate reverted back to above 40%. Therefore, SLXL1 plays a role in fertilization and its action can be blocked by its neutralizing antisera.

### Binding/penetration of spermatozoa to zona pellucida was inhibited by SLXL1 antiserum

As fertilization is a final readout of several earlier steps such as capacitation, acrosome reaction, and binding/penetration to zona pellucida of spermatozoa, it is necessary to check whether these steps could be affected by the SLXL1 antiserum. The capacitation of spermatozoa collected from the epididymis was induced by incubation in the HTF (Human Tubal Fluid) medium and were measured using the CASA system. As shown in Fig. 5B and 5C, neither the general motility nor the forward progression of spermatozoa was changed by the anti-SLXL1^1–155aa^ antiserum. In addition, acrosome reaction (AR) of spermatozoa is not changed significantly by the antiserum compared to the control preimmune serum group as shown by Fig. 5D.

**Figure 6:**
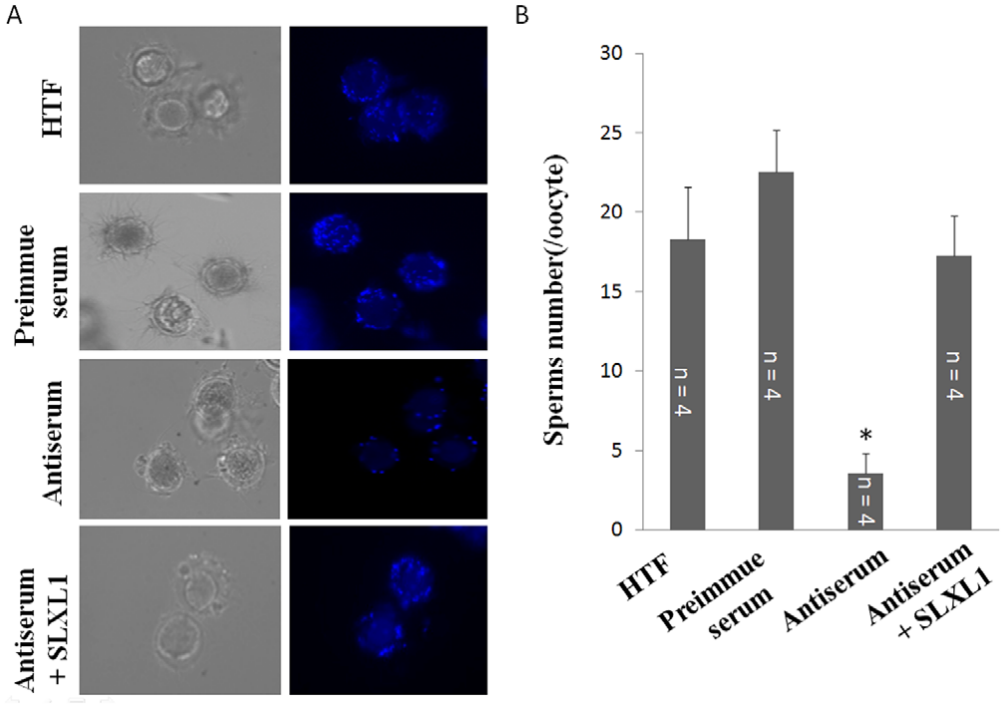
The inhibitory effect of SLXL1 antiserum on *in vitro* zona pellucida binding/penetration of spermatozoa. (A). Bright (left panel) and fluorescent (right panel) images of eggs and spermatozoa incubated with HTF, control, anti-SLXL1^1–155aa^ (antiserum) and anti-SLXL1^1–155aa^ plus SLXL1 (Antiserum+SLXL1). (B) Statistical analysis of binding/penetration of spermatozoa to oocytes. The number of bound spermatozoa on each egg was evaluated. *Denotes significantly different groups compared with control group (p<0.05).

The binding/penetration of spermatozoa to the zona pellucida of eggs was examined in the absence and presence of the anti-SLXL1^1–155aa^ antiserum. As shown by Fig. 6, while the control preimmune serum did not change the binding/penetration compared with the normal *in vitro* fertilization group (HTF), the antiserum reduced the binding/penetration significantly. The inclusion of SLXL1 recombinant protein in the antiserum treatment reversed the binding/penetration back to the normal level, indicating the inhibitory effect of the antiserum was specific to the endogenous SLXL1.

## Discussion

SLXL1 shares the highest amino acid identity with SLX, but its expression and potential role during spermatogenesis and/or fertilization has not been well addressed. The expression of Slxl1 mRNA and protein along with SLX in spermatogenesis have been reported in several studies [24], [25], but some questions remain open. First, what is the cellular and sub-cellular localization of SLXL1? The answer to this question was not straightforward. The main problem was that anti-SLXL1^1–155aa^ developed against full-length SLXL1 detected both itself and its close paralog SLX on Western blot while anti-SLXL1^65–155aa^, did not work on testis sections for immunostaining although it specifically recognized SLXL1 on Western blot. The cross-reaction of an anti-SLX antibody to SLXL1 on Western blot was also reported by others [26]. SLX was initially misidentified as being expressed in meiotic germ cells due to the use of an antibody that had not been well characterized [24], but has now been recognized as being expressed cytoplasmically in spermatids [25]. The signal detected by our anti-SLXL1^1–155aa^ was exclusively present in the acrosome of post-meiotic germ cells suggesting that on sections it detects SLXL1 but not SLX. On this basis, and given the interactions described below, it is evident that SLXL1 is an acrosomal protein, contrary to the expectation that it would be cytoplasmic based on its homology to SLX [26].

What does SLXL1 interact with in the acrosome? Based on the homology of SLXL1 and SLY, as well as the fact that SLY interacts with the acrosomal protein DKKL1, we hypothesized that SLXL1 also interacts with DKKL1. This turned out to be true as evidenced by IP assays using both over-expressed proteins in 293T cells and native proteins in testis lysates. Because SLY was reported to be a cytoplasmic protein and involved in regulating the expression of multiple genes [20], [21], the implication of its interaction with the acrosomal protein DKKL1 is unclear at the moment. In contrast, the interaction of SLXL1 with DKKL1 is easier to understand because they are both present in the acrosome. It will be interesting to examine whether SLX interacts with DKKL1 given its cytoplasmic localization. It is possible that DKKL1 interacts with different members of the same family as the acrosome forms in a step-wise manner. Therefore, it is critical to define the subcellular localization of these XLR proteins as well as to identify more interacting partners.

Perhaps the most important question related to its function is whether SLXL1 plays a role in fertilization. Although SLXL1 was suggested to be important for male fertility by one study [26], the simultaneous knockdown of both Slx and Slxl1 gene expression by siRNA made it impossible to evaluate the contribution of SLXL1. In the present study, we used an *in vitro* fertilization assay to investigate the role of SLXL1 using neutralizing antisera. The observation that the fertilization rate was significantly reduced by two antisera against SLXL1 (anti-SLXL1^1–155aa^ and anti-SLXL1^65–155aa^) but not by the antiserum against SLX (anti-SLX^95–212aa^) indicated convincingly that SLXL1 plays an important role in fertilization. The observations that neither the capacitation nor the acrosome reaction but the binding/penetration of spermatozoa was reduced by the antiserum further narrow down the time window during which SLXL1 executes its function. It is likely that SLXL1 is released from the acrosome and plays a role in the penetration of the spermatozoa into the oocytes, and during this time window the neutralizing antiserum has a chance to block its action. Alternatively, the antiserum may block the sperm-egg binding. If this is the case, it means that proteins released from the acrosome may stabilize the binding of spermatozoa to the eggs.

In conclusion, we have identified SLXL1 as an acrosome-associated member of the XLR family that plays an important role in fertilization probably involved in the penetration of spermatozoon through the zona pellucida or in the sperm-egg interaction. Given the different expression pattern and function of the characterized members of the family such as SYCP3, SLY, SLX, and SLXL1, it seems that XLR proteins may execute diverse functions during spermatogenesis.

## Materials and Methods

### Animals and Reagents

Adult ICR mice were obtained from the Experiment Animal Center, Chinese Academy of Sciences. New Zealand rabbits were purchased from the Experiment Animal Center, School of Medicine, Peking University. The animals were fed with normal chow and water in accordance with the NIH Guide for the Care and Use of Laboratory Animals. Ethical clearance was granted by the Animal Research Committee guidelines of the Institute of Zoology, Chinese Academy of Sciences (protocol study number 2004-35). Mouse anti–γH2AX (Ser139) was purchased from Millipore's Temecula. CA. Goat anti-DKKL1 was purchased from R&D systems, Inc. Mouse anti-GFP monoclonal antibody was purchased from Santa Cruz Biotechnology (Santa Cruz, CA). Mouse anti-Flag monoclonal antibody was purchased from Sigma (Sigma-Aldrich St. Louis, MO). Unless specified otherwise, the general reagents were purchased from Sigma and Invitrogen (Carlsbad, CA). Primers used in PCRs were synthesized by Invitrogen and listed in Table 1.

**Table 1:** Primer pairs used in the present study.

Name	Usage	Product Length	Sequence (5′-3′)
Slxl1-ad	RT-PCR	480 bp	Sense: ACA GAATTC ATG GCTCTTA AGAAACTGTAntisense: ACT CTCGAG TCTCAATTCA CCATCTAC
Slxl1-bc	RT-PCR	132 bp	Sense: ACA GAATTC ATGGAACAGCAAGATGGGAATAntisense: ACT CTCGAG CTCCCGTGTATCTCCAAC
Slxl1-ac	RT-PCR	321 bp	Sense: ACA GAATTC ATG GCTCTTA AGAAACTGTAntisense: ACT CTCGAG CTCCCGTGTATCTCCAAC
Slxl1-GST	Sub-Cloning into pGEX-4T-1	1–155AA	Sense: ACA GAATTC ATG GCTCTTA AGAAACTGTAntisense: ACT CTCGAG TCTCAATTCA CCATCTAC
Slxl1-C-GST	Sub-Cloning into pGEX-4T-1	65–155AA	Sense: ACA GAATTC ATGGAACAGCAAGATGGGAATAntisense: ACT CTCGAG TCTCAATTCA CCATCTAC
Slx-C-GST	Sub-Cloning intopGEX-4T-1	95–212AA	Sense: ACA GAATTC ATGGAACAGCAAGTTAGGAATAntisense: ACT CTCGAG TAATGTCTCTTCACCATC
Slxl1-GFP	Sub-Cloning intopEGFP-N1	1–155AA	Sense: ACT CTCGAG ATGGCTCTTA AGAAACTAntisense: ACA GAATTC TTCTCAATTCACCATCT

### RNA extraction and RT-PCR

Total RNAs from mouse tissues were extracted using Trizol solution following the standard protocol. cDNAs of Slx and Slx1 were cloned by standard RT-PCR using specific primers and sequencing confirmed.

### Recombinant protein expression and polyclonal antibody production

To produce glutathione S-transferase (GST) fusion protein, the mouse Slxl1 cDNA fragments (amino acids 1–155 aa and 65–155 aa) and Slx cDNA fragments (amino acids 95–212 aa) were subcloned into pGEX-4T-1 vector (Pharmacia Biosciences) using gene-specific primers (Table 1). Recombinant proteins were expressed in Escherichia coli strain BL21 and purified using GSTrap_5ml_FF columns (GE Healthcare) according to the manufacturer's instructions. About 0.5 mg of each recombinant GST fusion protein emulsified in Freund's complete adjuvant was injected subcutaneously into two New Zealand rabbits. Subsequently the rabbits were injected with 0.25 mg of the recombinant protein Freund's incomplete adjuvant every 2 weeks for 2 months. Finally, polyclonal antisera were recovered from the rabbit blood by standard methods.

### Western blot analysis

Mouse tissues and testes were homogenized in the RIPA lysis buffer (50 mM Tris, pH 7.4, 10 mM MgCl_2_, 150 mM NaCl, 1% NP-40, 1 mM sodium orthovanadate, 1 mM NaF). The protein concentration of extracts was determined using Bradford Reagent (BioRad) and were diluted in 6×SDS-loading buffer (125 mM Tris-HCl pH 6.8, 2% SDS, 20% glycerol, 0.2% bromophenol blue). Protein extracts from each tissue were run on 12% SDS-PAGE gels. Transfer of proteins in gels to Hybond-C membrane (Amersham) was performed using the Bio-Rad membrane transfer apparatus at 80 V for 2 h, and the membrane was then blocked in PBSA (5% milk powder and 0.1% Tween-20 in PBS) for 1 h at room temperature. First antibodies (anti-SLX^95–212aa^ diluted 1000 times, anti-SLXL1^1–155aa^ diluted 1000 times, anti-SLXL1^65–155aa^ diluted 1000 times and anti-GAPDH (diluted 2000 times) were added and incubation performed at 4°C overnight. The peroxidase-conjugated secondary antibody was used with a 2000-time dilution and signals were detected by chemiluminescence.

### Immunohistochemistry

Immunohistochemistry was performed following standard protocols. Briefly, 8 µm frozen sections of mouse testes were fixed immediately in 4% paraformaldehyde for 15 min at room temperature. The sections were washed with phosphate buffered saline (PBS) and blocked for 1 h in 5% bovine serum albumin in PBS. The section were then incubated with SLXL1^1–155aa^ antibody (diluted at 1∶100 in blocking buffer), SLX^95–212aa^ antibody (diluted at 1∶100), mouse anti-γH2AX (diluted at 1∶200) for 1 h at room temperature. The section was washed three times in PBS and incubated with secondary antibodies (FITC/TRITC-conjugated anti-rabbit, 1∶200, FITC- conjugated anti-mouse, 1∶200, Zhongshan Golden Bridge, Inc) for 1 h at temperature. The nuclei were stained with DAPI (0.5 µg/ml) for 10 min. After washing in PBS, the section was covered with slip and photographed by confocal microscopy. Rabbit pre-immune serum was used as negative control.

### Co-immunoprecipitation

The coding sequence of mouse Slxl1 cDNA and Dkkl1 cDNA were cloned into EGFP-N1 (BD Clontech) and pFlag-CMV-4 (Sigma). The plasmid constructs of SLXL1-GFP and Flag-DKKL1 were co-transfected into HEK293T cells using Lipofectamine 2000 according to the manufacturer's instructions (Invitrogen). pFlag-CMV-4 empty vector and Slxl1-GFP were transfected into HEK293T cells served as negative controls. Cells were solubilized in the RIPA lysis buffer 48 h after transfection and were incubated with Flag-conjugated sepharose beads overnight at 4°C. Beads and captured protein complexes(SLXL1-GFP and Flag-DKKL1) were washed 5 times. Beads were suspended in 6×SDS loading buffer and were analyzed by standard Western blotting with anti-GFP (1∶1000 dilution), anti-FLAG (1∶2000 dilution). Total protein lysates of the testis were incubated with anti-SLXL1^1–155aa^ antibody or rabbit preimmune serum for 2 h at 4°C, followed by incubation with protein A coated agarose beads overnight at 4°C. The agarose beads and captured protein complexes were washed 5 times in PBS and suspended in 6×SDS sample buffer for immunoblotting with anti-SLXL1^65–155aa^ (1∶1000 dilution) and anti-DKKL1 (1∶1000 dilution).

### In vitro fertilization

The female mice were superovulated and the stage MII oocytes were collected from mice oviducts as described [30]. Mouse spermatozoa from cauda epididymis were capacitated in HTF (Human Tubal Fluid) medium within 30 min, the spermatozoa (in drops of 50 µl with concentration of 5×10^4^/ml) were incubated for 20 min with the following 6 sets of reagents: i) HTF; ii) rabbit preimmune serum; iii) anti-SLXL1^1–155aa^; iv) anti-SLXL1^1–155aa^+SLXL1; v) anti-SLXL1^65–155aa^; vi) anti-SLX^95–212aa^. The treated spermatozoa (50 µl) was deposited into each medium drops (50 µl) containing 30∼35 mouse oocytes and incubated for 2 h. The unbound spermatozoa were washed away. To analyze the IVF rate, two pronuclei cells were examined 6 h after fertilization. The zygote cleavages were counted at 42 h.

### Capacitation analysis

Spermatozoa were obtained from the mouse epididymis and washed in PBS. Washed spermatozoa (5×10^7^/ml) were induced to capacitate in HTF medium or HTF medium with 0.3% SLXL1 antisera or 0.3% control preimmune serum by incubation for 2.5 h at 37°C under 5% CO_2_ as described [31]. To measure the motility of spermatozoa, a CASA system (Version.12 CEROS, Hamilton Thorne Research) was used with the following settings: minimal contrast, 50; minimal cell size, 4 pixels; and 60 frames acquired at a frame rate of 60 Hz. 400 individual spermatozoa in each sample were evaluated for general and progressive motility.

### Acrosome reaction analysis

Capacitated spermatozoa in each sample were incubated in the HTF medium supplemented with progesterone (P) to induce the acrosome reaction [32]. The samples were transferred to microslides, air dried and stained with Coomassie brilliant blue G-250 dye (CBB) in 10 ml of 3.5% (v/v) perchloric acid for 3 min [33]. The slides were rinsed with distilled water, the spermatozoa samples were observed under a phase contrast microscope. Acrosome reaction of the spermatozoa was examined from randomly selected fields of the slides until 400 spermatozoa had been examined.

### Zona pellucid binding/penetration assay

Oocytes were collected from ovaries and cumulus cells were removed with 0.01% (w/v) hyaluronidase [34]. About 8∼10 oocytes with intact zona pellucida (ZP) were incubated in a medium drop with HTF medium containing 0.3% pre-immune serum or 0.3% anti-SLXL1^1–155aa^. Fresh spermatozoa of cauda epididymis were capacitated in HTF medium for 30 min and deposited into each medium drop followed by incubation for another 30 min. Then oocyte-spermatozoon complex were washed thoroughly and fixed with 0.25% glutaraldehyde. In order to observe egg-spermatozoa binding/penetration, the complex were stained by 1 mg/ml Hoechst 33342 for 15 min and observed under a fluorescence microscope. The protocol followed that of a previous study with slight modifications [35].

### Statistical analysis

Statistical analysis was carried out using SPSS software (SPSS Statistics 17.0.1). Each experiment was repeated at least three times and differences were considered significant with p<0.05.
